# Impact of Different Promoters on Episomal Vectors Harbouring Characteristic Motifs of Matrix Attachment Regions

**DOI:** 10.1038/srep26446

**Published:** 2016-05-26

**Authors:** Xiao-Yin Wang, Jun-He Zhang, Xi Zhang, Qiu-Li Sun, Chun-Peng Zhao, Tian-Yun Wang

**Affiliations:** 1 Department of Biochemistry and Molecular Biology, Xinxiang Medical University, Xinxiang 453003, Henan, China

## Abstract

We previously demonstrated that the characteristic sequence of matrix attachment regions (MARs) allows transgenes to be maintained episomally in CHO cells. In the present study, six commonly used promoters from human cytomegalovirus major immediate-early (CMV), simian vacuolating virus 40 (SV40), Rous sarcoma virus, Homo sapiens ubiquitin C, phosphoglycerate kinase, and β-globin, respectively, were evaluated to determine their effects on transgene expression and stability in CHO cells stably transfected via the episomal vector harbouring characteristic MAR motifs. The CHO cells were transfected with vectors and then screened using G418, after which the stably transfected cells were split into two and further cultured either in the presence or absence of G418. Of the six promoters, the CMV promoter yielded the highest transgene expression levels and the highest transfection efficiency, whereas the SV40 promoter maintained transgene expression more stably during long-term culture than the other promoters did. The CMV and SV40 promoter-containing vectors were furthermore episomally maintained and conferred sustained eGFP expression in the cells even under nonselective conditions. On the basis of these findings, we conclude that the CMV promoter performs best in terms of yielding both high expression levels and high levels of stability using this episomal vector system.

Gene therapy involves the transfer of normal or therapeutic genes into target cells to correct genetic defects. Expression vectors play a key role in gene therapy and are classified as either viral or plasmid vectors based on content and the source of the DNA/RNA sequences. The gene transfer efficiency achieved with viral vectors is higher than that achieved with plasmid vectors; viral vectors are therefore more widely used^1^,^2^,^3^. Among viral vectors, retroviral vectors are the most commonly used because of the various advantages associated with their use,including the ability to efficiently infect a wide range of cell types from different animal species, the precise integration of genetic material carried by the vector into recipient cells, the lack of vector spread or production of viral proteins after infection, and the lack of toxicity of these viruses in infected cells. Transfection of retroviral vectors, however, depends on interactions between the vector and host cell membrane proteins, which could result in non-specific interactions and subsequent random integration into the host cell DNA; this, in turn, may cause serious complications such as insertional mutations or malignant transformation^4^,^5^. Stable transgene expression requires the vector to be integrated into the host cell chromosome^6^,^7^. Unlike retroviral vectors, conventionally used adenovirus vectors can be lost with cell division, and transgene expression is thus retained for a relatively short time^8^,^9^. Hybrid adenovirus vectors that combine genetic elements for stabilized transgene expression and high transduction efficiencies of adenoviruses can result in mitotically stable transgene expression, in which the somatic integration can be facilitated by integrases or transposases^10^,^11^. Plasmids offer some advantages compared with viral vectors; however, low transfection efficiency limits the use of plasmids in gene therapy. The expression of genes transfected via plasmids is, moreover, often transient.

Episomal vectors offer many advantages over integrating vectors as they eliminate non-specific integration into the host genome and with that eliminate the risk of transformation. Episomal vectors have the capacity for long-term persistence in mammalian cells, fulfilling the two main requirements of replication and segregation into daughter cells. Episomal vectors are based on origins of replication (OriPs) that derive from viral genomes such as those of human papovavirus BK, bovine papillomavirus type 1, and Epstein Barr virus (EBV). Since it was first described, the EBV-based episomal vector has been used repeatedly to transfer genetic material into various cell lines for functional analysis of genes^12^,^13^. The production of viral proteins in the host cell, however, poses serious risks for adverse responses if transferred in humans^14^.

Vectors harbouring matrix attachment regions (MARs) can replicate episomally in an eukaryotic cell and do not require any virally encoded trans-acting factors for replication. pEPI-1 is one such episomal system that has been suggested to have considerable potential for biotechnological applications; its design is based on the SV40 OriP. This system also exploits the human β-interferon scaffold/matrix attachment region (S/MAR)^15^,^16^. Active transcription upstream of the S/MAR running into this sequence is required and is probably sufficient for episomal replication^17^. Episomal vectors have been used in the treatment of retinal diseases and tumors^18^,^19^,^20^, whereas MARs have been shown to mediate episomal replication of lentiviral vectors in Chinese hamster ovary (CHO) cells and to drive efficient transgene expression^21^,^22^. Voigtlander *et al*. developed a hybrid-vector system that synergizes high-capacity adenoviral vectors for efficient delivery and the S/MAR-based pEPito plasmid replicon for episomal persistence^23^.

In our previous study, an episomal vector harbouring a 367-bp DNA sequence comprising a characteristic MAR motif was constructed^24^; however, the expression levels and transgene copy number were limited. Transgene expression levels and stability are determined by the composition of the plasmid vector, including promoters, polyadenylation signals, and other expression elements. Haase *et al*. showed that the human AFP-promoter in combination with the hCMV enhancer element acts as a valuable tissue-specific promoter for targeting hepatocellular carcinomas with a non-viral gene delivery system, thereby yielding higher tissue-specificity with less undesired side effects^20^. It has been demonstrated that genomic cis-acting sequences, including a ubiquitous chromatin-opening element (UCOE) and an insulator sequence (cHS4), mediated enhanced transgene expression and establishment efficiency^25^.

Human cytomegalovirus major immediate-early (CMV), Rous sarcoma virus (RSV), Homo sapiens ubiquitin C (UBC), simian vacuolating virus 40 (SV40), phosphoglycerate kinase (PGK), and β-globin promoters are the common promoters used in mammalian cells such as CHO cells^26^,^27^,^28^. Although there are some reports on the use of these promoters in recombinant CHO cells, there are no reports on the effect of these promoters on episomal vectors. In the present study, the performance of CMV, RSV, SV40, UBC, PGK, and β-globin promoters in the episomal vector were evaluated in terms of transgene expression level and stability in CHO cells. The findings reported here will be helpful to researchers designing episomal vectors in terms of optimal promoter selection for high expression and long-term stability of transgenes.

## Results

### Positive recombinant protein expression rate

**Figure 1:**
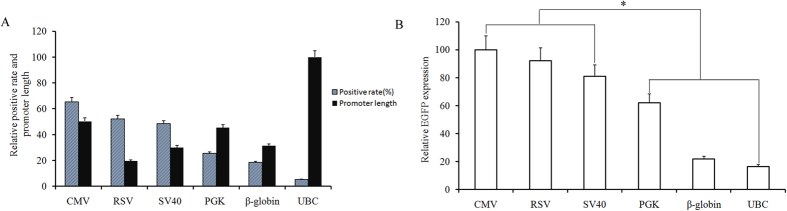
Positive recombinant protein expression rates, relative promoter lengths, and eGFP expression levels in transfected CHO cells. Six different vectors were each transfected into CHO cells and the number of cell expressing the eGFP gene was determined by flow cytometry after 48 h transfection. Relative expression rate was normalized for promoter size (**A**); Impact of MAR in combination with different promoters on eGFP gene expression levels in transfected cell pools (**B**). Three stably transfected pools were generated for each vector. Cells were collected and measured for the eGFP MFI with the FACS Calibur. Mean values differed significantly (*P < 0.05) between the vectors containing the CMV, RSV, or SV40 promoter and those containing the PGK, β-globin, or UBC promoter.

Each of the six constructed plasmids was transfected into CHO cells, and for each plasmid the number of cells expressing the eGFP gene was determined by flow cytometry 48 h after transfection. The positive rate of recombinant protein expression was found to be highest for the plasmids containing the CMV promoter (65.32%), followed by those containing the RSV (52.23%), SV40 (48.36%), and PGK (25.35%) promoters. The plasmids containing the β-globin and UBC promoters yielded the lowest positive rates (18.37% and 15.27%, respectively; Fig. 1A). The CMV, RSV, SV40, PGK, β-globin, and UBC promoters were 589 bp, 229 bp, 351 bp, 555 bp, 366 bp, and 1168 bp in length (Supplementary Fig. S1). Setting the length of the UBC promoter to a value of 100 and normalizing the other promoter lengths to this, promoter length was ruled out as a factor determining positive rate: the CMV promoter was the second longest, only shorter than that of UBC, but showed the highest activity (Fig. 1A).

### Recombinant protein expression levels

The eGFP protein levels (median fluorescence intensity, MFI) were measured using flow cytometry. Cells transfected with the vector containing the CMV promoter exhibited the highest expression levels, followed by those transfected with RSV and SV40 promoter-containing vectors. The eGFP expression levels were low in cells transfected with PGK, UBC, and β-globin promoter-containing vectors. When the eGFP expression level under the CMV promoter was considered 100, the expression levels under the RSV, SV40, PGK, β-globin,and UBC promoters were 92.21, 81.13, 62.14, 21.78, and 16.72, respectively. Expression under the CMV, RSV, and SV40 promoters was significantly higher than that under the PGK, β-globin, and UBC promoters (*P* < 0.05). The highest activity was thus exhibited by the CMV promoter, and the MFI in CMV promoter-transfected cells was 5.87-fold that in UBC promoter-transfected cells (Fig. 1B).

### Gene copy number analysis

**Figure 2:**
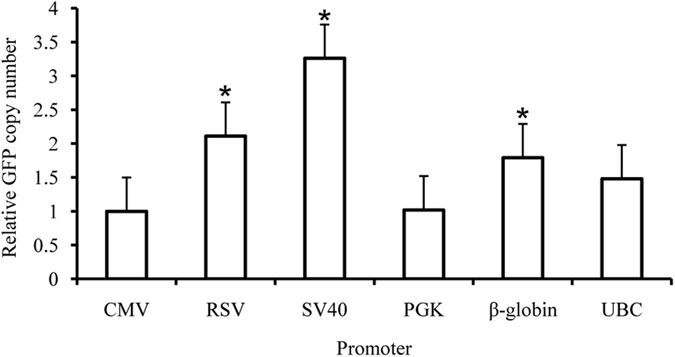
Relative gene copy numbers in transfected CHO cells. The relationship between transgene expression levels and copy number was assessed by quantitative PCR analysis (n = 3). Mean values differed significantly (*P < 0.05) between the vectors containing the CMV promoter and those containing the RSV, SV40, or β-globin promoter.

To investigate the relationship between eGFP expression levels and copy number for the episomal vectors in CHO cells, fluorescence quantitative PCR analysis was performed. The results of this analysis revealed that the gene copy number for the CMV promoter was lower than that for the SV40, RSV, and β-globin promoters (*P* < 0.05, Fig. 2). The eGFP expression levels determined by flow cytometry, however, were higher for the CMV promoter than for the other five promoters, suggesting that the levels of eGFP expression were not related to gene copy number, but rather to the promoter activity.

### Long-term stability of recombinant proteins

**Figure 3:**
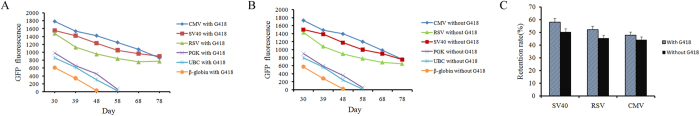
Long-term recombinant protein expression stability in transfected CHO cells. Stably transfected CHO cells were selected after transfection by the addition of G418. The cells were split in two and further cultured either in the presence or the absence of G418 before flow cytometric analysis. The eGFP expression retention was calculated as the ratio of the MFI at the end of stability testing to the MFI at the start of stability testing. Relative changes in eGFP expression level at different days post-transfection in the presence (**A**) or absence (**B**) of selection pressure. Retention of eGFP expression levels in cells transfected with SV40, RSV, and CMV promoter-containing vectors (**C**; n = 3).

**Figure 4:**
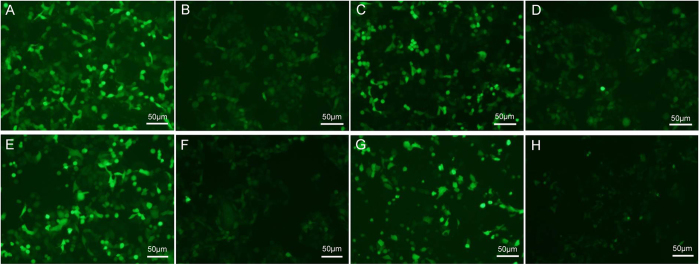
Fluorescence microscopy of eGFP gene in transfected CHO cells grown in the presence or absence of G418 selection pressure. CHO cells transfected with (**A**) the CMV promoter-containing vector at day 30 post-transfection in the presence of selection pressure; (**B**) the CMV promoter-containing vector at day 78 post-transfection in the presence of selection pressure; (**C**) the SV40 promoter-containing vector at day 30 post-transfection in the presence of selection pressure; (**D**) the SV40 promoter-containing vector at day 78 post-transfection in the presence of selection pressure; (**E**) the CMV promoter-containing vector at day 30 post-transfection in the absence of selection pressure; (**F**) the CMV promoter-containing vector at day 78 post-transfection in the absence of selection pressure; (**G**) the SV40 promoter-containing vector at day 30 post-transfection in the absence of selection pressure; (**H**) the SV40 promoter-containing vector at day 78 post-transfection in the absence of selection pressure.

The stably transfected CHO cells were cultured either in the presence or absence of G418 selection pressure and MFI was measured in the cells to assess the stability of the expressed recombinant protein at days 30, 39, 48, 58, 68, and 78 post-transfection. In all stably transfected CHO cells, eGFP levels decreased gradually over time. eGFP expression under the CMV promoter was the highest before day 70 post-transfection; however, this level was lower than that that for the SV40 promoter at day 78 post-transfection (Fig. 3A,B). In contrast, none of the clones generated using UBC, PGK, and β-globin promoters were stable: only 2.91%, 6.43%, and 5.73%, respectively, of the initial levels of expression were retained in these clones (Fig. 3A,B). Expression levels did not differ significantly between clones cultured in presence of G418 selection pressure compared with those cultured in the absence of G418. The most stable expression was achieved under the SV40 promoter: CHO cells transfected with the SV40 promoter-containing vector maintained 68.04% and 58.31% of the original expression levels by days 58 and 78 post-transfection, respectively (Fig. 3C). In agreement with the MFI results, eGFP gene expression observed by fluorescence microscopy was also evident at days 30 and 78 post-transfection in cells cultured under selection pressure (Fig. 4A–D). The eGFP gene expression was also well maintained at days 30 and 78 post-transfection in the absence of selection pressure (Fig. 4E–H).

### Plasmid rescue assay

**Figure 5:**
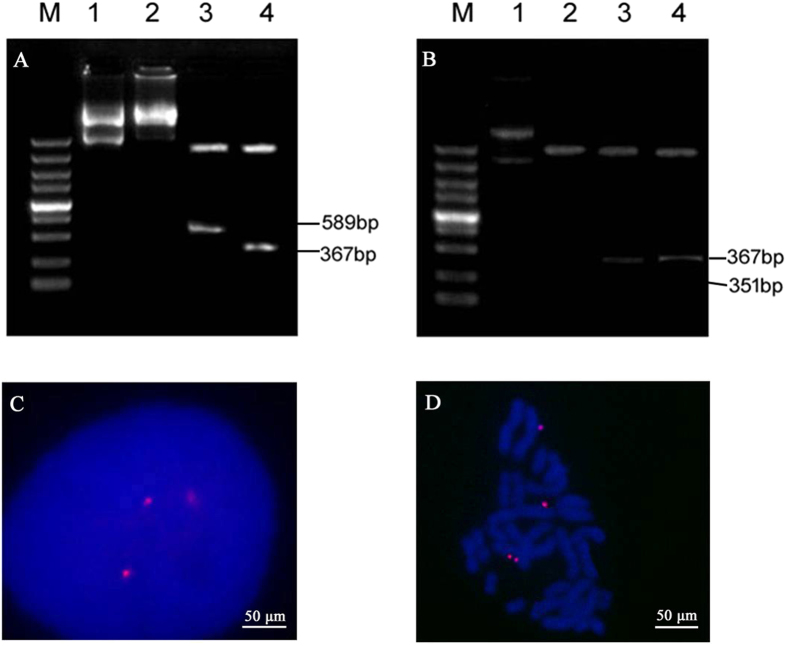
Determination of episomal *vs.* integrated status of plasmids in transfected CHO cells. (**A**,**B**) DNA was extracted from CHO cells transfected with vectors containing the CMV and SV40 promoters, after which the extracted DNA was used to transform DH5α *E. coli* cells by electroporation. Transformants were selected and plasmid DNA was prepared and subjected to restriction analysis. Agarose gel electrophoresis of DNA extracted from CHO cells transfected with (**A**) the CMV promoter-containing vector and (**B**) the SV40-containing vector: M, DL5000; Lane 1, Extracted plasmid; 2, *Kpn* I digestion; 3, *Ase* I/*Nhe* I digestion; 4, *Kpn*I/*Bam*HI digestion. (**C**,**D**) Association of the episome with metaphase chromosomes from CHO cells transfected with vectors containing the CMV promoters. CHO cells transfected with plasmid were analysed by DNA FISH to assess whether the vectors were present as integrated copies. The episome (red) was visualized by eGFP FISH. Vector molecule distribution was monitored in post-mitotic nuclei of dividing cells (**C**) and episome localization was determined by FISH on spreads of metaphase chromosomes (**D**).

Hirt DNA extraction was performed to recover extrachromosomal DNA from CHO cells transfected with vectors containing the CMV and SV40 promoters. The plasmid rescue assay was performed as described previously^15^,^24^ and the rescued plasmids were identified by digestion with selected restriction enzymes: *Ase* I/*Nhe* I digestion was expected to yield 589 bp and 351 bp fragments, whereas digestion with *Kpn* I/*Bam* HI was expected to yield a 367 bp fragment. The plasmids rescued from cells transfected with the CMV and SV40 promoter-containing vectors yielded the expected fragments (Fig. 5A,B), indicating that these two vectors existed episomally in the CHO cells and were not integrated into the host cell genomic DNA.

### Fluorescence *in situ* hybridization (FISH) analysis

**Figure 6:**
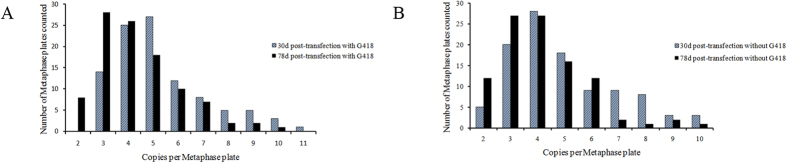
Gene copies per metaphase plate as determined by FISH analysis. FISH analysis was performed on CHO cells stably transfected with CMV promoter-containing vector at days 30 and 78 post-transfection in the presence (**A**) or absence (**B**) of selection pressure.

We previously reported that vectors mediated by characteristic motifs of MARs can exist episomally in stably transfected CHO cells^24^. To demonstrate episomal vector replication, FISH analysis was performed on spread chromosomes of CHO cells transfected with the CMV promoter-containing vector either in the presence or the absence of G418 selection pressure. The FISH analysis revealed that the observed mitotic stability of the vector was a result of the vector existing episomally on metaphase chromosomes—a MAR-mediated state—thus preventing vector integration into the genomic DNA (Fig. 5C,D). One-hundred metaphase plates were analysed by FISH for each vector. The average copy numbers determined from this analysis were 5.31 ± 1.88 and 4.36 ± 2.60 in CHO cells at days 30 and 78 post-transfection, respectively, in the presence of selection pressure (range, 2–11 copies per cell; Fig. 6A). At 30 and 78 days post-transfection, the average vector copy numbers per cell were 4.88 ± 1.96 and 4.15 ± 1.61 in CHO cells cultured in the absence of selection pressure, respectively (range, 2–10 copies per cell; Fig. 6B). Whether or not the vector has a preferred binding site cannot be confirmed; however, no such site was observed. There was no significant difference in copy numbers between the presence and absence of selection pressure. These findings demonstrate that the vector can replicate episomally without significant copy number loss with cell division, and that this capacity is not compromised by the absence of selection pressure.

### Analysis of transcription factor regulatory elements (TFREs) on transgene expression

Promoter activity relates to transcription factor binding sites and transcription factor regulatory elements (TFREs). The distribution of nine TFREs (SP1, NFkB, E-box, AP1, CREB, BEDF, E4FF, CEBP, and OCT; Supplementary Dataset) was therefore assessed for the six promoters. The SP1, NFkB, AP1, and OCT TFREs were abundant in both the CMV and the SV40 promoters (Table 1). The RSV promoter was abundant in the AP1 and OCT TFREs, but did not contain SP1 and NFkB TFREs. The findings led us to conclude that the NFkB, Oct1, and AP-1 TFREs contribute to promoter activity, whereas the SP1 TFRE plays a role in maintaining transgene expression.

## Discussion

Long-term gene expression is dependent on efficient vector retention, which can be achieved by the use of a replicating episomal vector. Such vectors offer several advantages over integrating vectors by maintaining the transgene in an extrachromosomal state. Transgene expression levels and stability are influenced by various components of the plasmid vector including the promoter. In this study, the relative activities of six commonly used promoters (CMV, RSV, SV40, UBC, PGK, and β-globin) in an episomal vector designed using characteristic MAR motifs were assessed in CHO cells. The episomal vector was first discovered in CHO cells^15^, and hence CHO cells were used in this study. The highest recombinant protein (eGFP) expression levels were obtained under the CMV promoter, followed by the RSV and SV40 promoters. The highest transection efficiency, too, was achieved with the vector containing the CMV promoter. The CMV promoter, which is currently used to drive the expression of many biopharmaceutical products, is considered a potent promoter and is a highly complex element evolved by the virus to enable infection of many mammalian host cells^29^.

The delivery of vectors containing large DNA fragments by non-viral means has proven to be inefficient. Among the six promoters tested here, the RSV promoter was the shortest in length, followed by the β-globin and SV40 promoters. The highest positive eGFP gene expression rate was achieved with the CMV promoter-containing vector, suggesting that transgene expression was not only affected by promoter length, but also by promoter composition, cell type, cell state etc. The activity of different promoters thus depends not only on a single factor such as the length, but also on other factors influencing the performance of episomal vectors, such as genomic cis-acting sequences and cell line differences. The UCOE and cHS4 elements have been shown to enhance transgene expression and establishment efficiency^25^. Long-term gene expression from the pEPI vector has been demonstrated in K562 human hematopoietic progenitor cells, CHO cells, HeLa cells, human hepatoma Huh7 cells, and HaCaT cells^12^,^30^,^31^,^32^,^33^. In contrast, in the MEL murine erythroleukemia cell line, transgene expression can be silenced through histone deacetylation, despite episomal persistence of the vector^32^.

Transcription factor binding sites or TFREs can influence promoter activity. The TFREs of the six promoters used in this study were analysed, and it was shown that the NFkB TFRE was abundant in the CMV and SV40 promoters, but not in the RSV promoter. E-box TFREs, on the other hand, were only identified in the UBC and β-globin promoters. The Sp1 and AP-1 transcription factors are often final targets of signal-transducing kinase cascades, and upon phosphorylation, become activated and bind to their respective target promoters, thereby triggering the expression of the corresponding genes^34^,^35^. The CMV, RSV, and SV40 promoters were found to contain a few Oct1 and AP-1 TFREs, whereas pGK and UBC promoters, which were less active, contained fewer of these TFREs. These findings led us to speculate that the NFkB, Oct1, and AP-1 TFREs contribute to promoter activity in episomal vectors.

An ideal episomal vector for functional genetics or gene therapy applications should allow long-term expression of the delivered transgene at close to physiological levels. The differences between the eGFP levels observed for the CMV, SV40, and RSV promoters suggest that the SV40 promoter is more resistant to transcriptional silencing. Although the CMV promoter is a strong promoter, it is intrinsically susceptible to silencing, resulting in reduced productivity over time during long-term culture^28^. Transcriptional silencing can be caused by DNA methylation^36^,^37^,^38^. The CMV promoter contains 32 CG dinucleotides, whereas the SV40 promoter contains only 10 CG dinucleotides (Supplementary Fig. S2). This may be one reason for the greater resistance to DNA methylation-induced transcriptional silencing exhibited by the SV40 promoter compared with the CMV promoter. The CMV promoter in an episome is reportedly not subject to silencing by cytosine methylation, thus allowing long-term expression of the transgene in the absence of selection^39^. In this study, we identified six and five SP1 TFREs in the SV40 and CMV promoters, respectively. SP1 TFREs have been previously proposed to inhibit DNA methylation^40^,^41^. In contrast, the RSV promoter contains nine CG dinucleotides, but no SP1 TFREs. These findings suggest that the retention of transgene expression is influenced by both DNA methylation and TFREs.

To trace the presence and localization of the six different vectors in the transfected CHO clones, extrachromosomal DNA was extracted and a plasmid rescue assay was performed. Owing to the low copy number of episomal vectors in CHO cells, a large number of cells (10^9^) was used in the extrachromosomal DNA extraction by the Hirt method, after which the extracted plasmids were rescued through transfection-competent *Escherichia coli* cells. The plasmids were finally identified by digestion with selected restriction enzymes. The DNA fragment pattern obtained from the restriction enzyme digests clearly demonstrated the episomal state of the constructed vectors—the vectors were not integrated into the host cell genomic DNA. The episomal status was further confirmed by FISH analysis of cells at different days post-transfection following culture in the presence or absence of selection pressure: the transgene was found to exist episomally on metaphase chromosomes not integrated in the genomic DNA. The gene copy number furthermore did not differ significantly between cells cultured in the presence *vs.* the absence of selection pressure.

Some epigenetic regulators such as special (A + T)-rich binding protein 1 (SATB1), nuclear matrix protein 4 (NMP4), or CTCF, can be bound by MAR^42^,^43^; and MAR-binding proteins may also recruit histone acetyltransferases and ATP-dependent chromatin remodelling complexes, thereby increasing gene expression^44^. Arope *et al*. showed that the AT-rich core elements of MAR primarily exerts an anti-silencing effect, whereas the transcriptional augmentation effect results more prominently from the transcription factor binding motifs present on the flanking sequences^45^. To design an optimal vector for gene therapy, however, it is essential to understand how these regulatory elements work in co-ordination in the vector. It has been suggested that mitotic stability is provided by the specific interaction of an episomally replicating vector with components of the nuclear matrix. Jenke *et al*. found that the vector binds exclusively to hnRNP-U/SAF-A, a multifunctional scaffold/matrix specific factor^46^. Compared with the episomal vector pEPI-1 based on MARs, the episomal vector used in this study is short, easy to transform, and yields higher expression levels and stability.

In this study, six promoters’ activities were investigated in an episomal vector system and of the six promoters, the CMV promoter yielded the highest transgene expression levels and the highest transfection efficiency. The SV40 promoter, on the other hand, maintained transgene expression more stably during long-term culture than the other five promoters. The findings of this study furthermore demonstrated that, in the presence or absence of selection pressure, the vector can replicate episomally without significant copy number losses with cell division.

## Methods

### Vector construction

**Figure 7:**
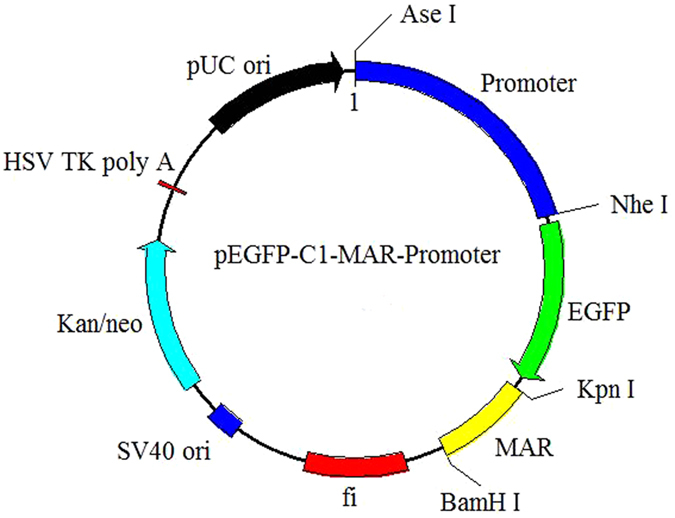
Schematic illustration of expression vectors containing different promoters. The vectors were derived from the commercial pEGFP-C1 plasmid^24^. A MAR sequence comprising a characteristic MAR motif from the human IFN-β gene was cloned into the multiple cloning site (MCS) of pEGFP-C1 resulting in the vector pEGFP-C1-MAR-promoter. The Neo/Kan gene is driven by dual promoters to confer kanamycin resistance in bacteria and G418 resistance in mammalian cells. *Ase*I and *Nhe*I restriction sites are indicated. SV40, simian virus 40; ori, origin of replication; HSV, herpes simplex virus. The CMV promoter was excised and replaced by different promoters (RSV, UBC, SV40, PGK, and β-globin promoters) to produce the vectors used in this study.

Primers (Table 2) were designed based on the sequences of the six promoters of interest (Supplementary Fig. S1). The RSV and SV40 promoters were artificially synthesized by Sangon Biotech Co., Ltd (Shanghai, China), whereas the PGK, UBC, and β-globin promoters were generated using PCR methods. To achieve directional cloning, an *Ase*I/*Nhe*I enzyme site was introduced at the 5′ ends of the primers. For PCR, the following cycling parameters were used: four cycles of 95 °C for 3 min, 94 °C for 40 s, 60–56 °C for 30 s, 72 °C for 40 s, followed by 20 cycles at 55 °C, and a final step at 72 °C for 3 min. The PCR products were recovered and their sequences were confirmed before they were digested with *Ase*I/*Nhe*I. The products were then ligated into the pEGFP-C1-MAR vector to produce the six different vectors, each with a different promoter^24^. The vector identities were verified by enzyme digestion and sequencing. Agarose gel electrophoresis was performed as described by Zhang *et al*.^47^. The constructs are described schematically in Fig. 7.

### Cell culture and transfection

CHO cells (provided by the Institute of Laboratory Animal Sciences, Beijing, China) were cultured in Dulbecco’s Modified Eagle’s Medium (Gibco, Carlsbad, CA) supplemented with 10% foetal bovine serum (Gibco, Grand Island, NY) in a humidified incubator at 37 °C with 5% CO_2_. The cells were plated at a concentration of 3 × 10^6^ cells/well in six-well plates. At 70% confluence, the cells in each well were transfected with Lipofectamine^®^ 2000 Transfection Reagent (Thermo Fisher Scientific,MA,USA) according to the manufacturer’s instructions. Stably transfected cells were selected 48 h after transfection by the addition of Geneticin (G418, Invitrogen,MA,USA) to the culture medium at a concentration of 800 mg/ml. At week 2 post-transfection, the stably transfected cells were split in two and further cultured either in the presence or absence of G418 (400 mg/ml). At 70–80% confluence, the cells were collected for analysis.

### Flow cytometry

To determine the proportion of eGFP-positive cells and thus the eGFP gene expression levels, 1 × 10^4^ ~ 1 × 10^6^ CHO cells were collected and analysed by flow cytometry 48 h after transfection. The results were analysed using the FlowJo software 7.6 (Tree Star, Ashland, OR) and untransfected cells were used as the negative control. In the analysis, the eGFP-positive cells (M1) were set such that the number of cells in the negative control M1 area were <0.1%, where the positive expression rate was calculated as the cells in the M1-negative control sample as a percentage of the cells in the M1 area. Three stably transfected pools were generated for each vector. To characterize each stably transfected pool, cells (4 × 10^5^ cells/ml) were seeded into the wells of 6-well plates 2 weeks after G418 screening. The eGFP expression levels in the cells were directly determined using a FACS Calibur cytometer (Becton Dickinson, New Jersey, USA). A total of 100,000 fluorescent events were acquired using a 530/15 bandpass filter for the green fluorescent signal acquired with an emission wavelength of 530 nm. The median fluorescence intensities (MFIs) for the vectors were also measured.

### Stability testing

The CHO cells stably transfected with the vectors containing the CMV, RSV, and SV40 promoters were passaged in 6-well plates and further cultured. The MFI for each vector type was measured using the FACSCalibur cytometer and the retention of eGFP expression for each vector was calculated as the ratio of the MFI at the end of stability testing to the MFI at the start of stability testing.

### Plasmid rescue assay

Episomal DNA from the CHO cells transfected with the vectors containing the CMV and SV40 promoters was prepared according to a modified version of the method described by Hirt *et al*.^24^,^48^. Briefly, cells were collected by centrifugation, suspended at a density of 1 × 10^9^ cells, washed in TEN buffer (10 mM Tris-HCl [pH 7.5], 1 mM EDTA, 150 mM NaCl), resuspended in 1.5 ml TEN and 1.5 ml 2× HIRT buffer (1.2% SDS, 20 mM Tris-HCl [pH7.5], 20 mM EDTA), and incubated for 20 min at room temperature. NaCl (1 M final concentration) was added to lyse the cells, after which the cells were incubated overnight at 4 °C. After centrifugation for 30 min at 15,000 × *g*, the supernatant was extracted twice using a 1:1 phenol:chloroform solution and twice using chloroform. Nucleic acids were precipitated and resuspended in 20 ml TE (10 mM Tris, 1 mM EDTA) with 50 mg/ml RNAse A. Ten microliters of the HIRT extract was used to transform DH5α *E. coli* cells by electroporation. The *E. coli* transformants were selected using plates containing 30 mg/ml kanamycin. Plasmid DNA was prepared from randomly picked resistant colonies and was subjected to restriction analysis by digestion with *Kpn*I/*BamH* I and *Ase*I*/Nhe*I.

### Fluorescence quantitative PCR

Relative eGFP gene copy numbers were determined using fluorescent quantitative PCR. Episomal DNA was extracted from the cells as described for the plasmid rescue assay. The following primers were used in the subsequent PCR analysis: 5′-GCTGGTTTAGTGAACCGTCAG-3′ (eGFP forward), 5′-AGGTGGCATCGCCCTCGCCC-3′ (eGFP reverse), 5′-GATGGGGTACCCTTCATCC-3′ (G6PDH forward), and 5′-GCTCTGACTCCTCAGGGTTG-3′ (G6PDH reverse). Ready-to-use “hot-start” FastStart DNA Master PLUS SYBER Green I fluorescent reaction mix (Roche,Switzerland) and an ABI 7500 SYBER Fluorescence quantitative PCR instrument (Applied Biosystems, Foster City, CA) were used for PCR reactions, which were performed with 40 cycles using the manufacturer-recommended parameters. Relative eGFP copy numbers were calculated using the 2^−ΔΔCt^ method. All the experiments were repeated three times.

### FISH analysis

The FISH analysis was performed as described by Lin *et al*.^24^. Cells grown in the presence or absence of selection pressure were collected at days 30 and 78 post-transfection. eGFP was used as a probe and was labelled using either Biotin- or Digoxigenin-Nick translation kits (Roche, Mannheim, Germany). Samples were counterstained with 1 μg/ml 4′,6′-diamidino-2-phenylindole before being analysed using a Leica DMRB fluorescence microscope with a Leica DC 300 f camera. Approximately 50 fields were observed and mean copy numbers were calculated. One hundred metaphase plates were analysed by FISH for each vector type.

### Bioinformatics analysis

Specific transcription factor binding sites were identified using MatInspector software (http://www.genomatix.de/products/index.html)^49^, whereas CpG islands were analysed using CPGPLOT^50^.

### Statistical analysis

All experimental data ware analysed using SPSS 18.0 software (SPSS Inc., Chicago, IL). Data are reported as mean ± standard deviation. Comparisons between different groups were analysed using single factor analysis of variance, and *t*-tests were used for pairwise comparisons. Differences with *P* values < 0.05 were considered statistically significant.

## Additional Information

**How to cite this article**: Wang, X.-Y. *et al*. Impact of Different Promoters on Episomal Vectors Harbouring Characteristic Motifs of Matrix Attachment Regions. *Sci. Rep.* **6**, 26446; doi: 10.1038/srep26446 (2016).

## Supplementary Material

Supplementary Information

Supplementary Dataset

## Figures and Tables

**Table 1:** Locations of various transcription factor binding motifs within the six promoters.

Promoter	CMV	PGK	RSV	SV40	Ubc	β-globin	Strand
SP1	0	4	0	0	5	0	+
	5	1	0	6	2	1	−
NFkB	4	1	0	0	0	1	+
	0	1	0	2	0	0	−
E-box	0	0	0	0	1	0	+
	0	0	0	0	1	1	−
AP1	4	2	2	4	1	2	+
	3	1	0	3	2	1	−
CREB	13	1	0	0	7	0	+
	9	5	0	0	8	0	−
BEDF	0	2	0	0	3	0	+
	2	1	0	4	0	0	−
E4FF	4	1	0	0	2	0	+
	5	0	0	0	2	0	−
CEBP	0	1	1	2	1	1	+
	1	0	2	0	0	3	−
OCT	1	0	2	4	1	1	+
	2	0	1	3	0	2	−

**Table 2:** Primers used in this study.

Promoter	Sequence (5′-3′)	Length(bp)
UBC	F: 5′-GGCCTCCGCGCCGGGTTTTGGC-3′R: 5′- TTTACTAGTCTAACACTGAAAA-3′	1168
PGK	F: 5′-TTGGGGTTGCGCCTTTTCCAAG-3′R: 5′-GCGCACCGTGGGCTTGTACT-3′	555
β-globin	F: 5′-GCTTTGCTTC TCAATTTCTT-3′R: 5′-CAACACAAACTATGTCAGAAG-3′	366
