# Supplementary material for: Impact of Different Promoters on Episomal Vectors Harbouring Characteristic Motifs of Matrix Attachment Regions
Source: Sci Rep. 2016 May 26;6:26446. doi: 10.1038/srep26446 (PMC4881036; doi:10.1038/srep26446)
Supplement: Supplementary Information [file srep26446-s1.pdf]

Impact of Different Promoters on Episomal Vectors harboring Characteristic Motifs of Matrix Attachment Regions

Running title: Promoters and episomal vectors

Xiao-Yin Wang, Jun-He Zhang, Xi Zhang, Qiu-Li Sun, Chun-Peng Zhao, Tian-Yun Wang*

Department of Biochemistry and Molecular Biology, Xinxiang Medical University, Xinxiang 453003, Henan, China

*Corresponding author:

Tian-Yun Wang

Department of Biochemistry and Molecular Biology,

Xinxiang Medical University,

Jinsui Road,

Xinxiang 453003,

Henan,

China

Tel: +86 373 3831899

E-mail: wtianyuncn@126.com

Fig. S1 Sequence of primers used in this study

CMV promoter sequence (589bp)

TAGTTATTAATAGTAATCAATTACGGGGTCATTAGTTCATAGCCCATATATGGAGTT
CCGCGTTACATAACTTACGGTAAATGGCCCGCCTGGCTGACCGCCCAACGACCCCCG
CCCATTGACGTCAATAATGACGTATGTTCCCATAGTAACGCCAATAGGGACTTTCCA
TTGACGTCAATGGGTGGAGTATTTACGGTAAACTGCCCACTTGGCAGTACATCAAGT
GTATCATATGCCAAGTACGCCCCCTATTGACGTCAATGACGGTAAATGGCCCGCCTG
GCATTATGCCCAGTACATGACCTTATGGGACTTTCCTACTTGGCAGTACATCTACGT
ATTAGTCATCGCTATTACCATGGTGATGCGGTTTTGGCAGTACATCAATGGGCGTGG
ATAGCGGTTTGACTCACGGGGATTTCCAAGTCTCCACCCCATTGACGTCAATGGGAG
TTTGTTTTGGCACCAAAATCAACGGGACTTTCCAAAATGTCGTAACAACTCCGCCCC
ATTGACGCAAATGGGCGGTAGGCGTGTACGGTGGGAGGTCTATATAAGCAGAGCTGG
TTTAGTGAACCGTCAGATC

Ubc promoter sequence (1168 bp)

GGCCTCCGCGCCGGGTTTTGGCGCCTCCCGCGGGCGCCCCCCTCCTCACGGCGAGCG
CTGCCACGTCAGACGAAGGGCGCAGGAGCGTCCTGATCCTTCCGCCCGGACGCTCAG
GACAGCGGCCCGCTGCTCATAAGACTCGGCCTTAGAACCCCAGTATCAGCAGAAGGA
CATTTTAGGACGGGACTTGGGTGACTCTAGGGCACTGGTTTTCTTTCCAGAGAGCGG
AACAGGCGAGGAAAAGTAGTCCCTTCTCGGCGATTCTGCGGAGGGATCTCCGTGGGG
CGGTGAACGCCGATGATTATATAAGGACGCGCCGGGTGTGGCACAGCTAGTTCCGTC
GCAGCCGGGATTTGGGTCGCGGTTCTTGTTTGTGGATCGCTGTGATCGTCACTTGGT
GAGTAGCGGGCTGCTGGGCTGGCCGGGGCTTTCGTGGCCGCCGGGCCGCTCGGTGGG
ACGGAAGCGTGTGGAGAGACCGCCAAGGGCTGTAGTCTGGGTCCGCGAGCAAGGTTG
CCCTGAACTGGGGGTTGGGGGGAGCGCAGCAAAATGGCGGCTGTTCCCGAGTCTTGA
ATGGAAGACGCTTGTGAGGCGGGCTGTGAGGTCGTTGAAACAAGGTGGGGGGCATGG
TGGGCGGCAAGAACCCAAGGTCTTGAGGCCTTCGCTAATGCGGGAAAGCTCTTATTC
GGGTGAGATGGGCTGGGGCACCATCTGGGGACCCTGACGTGAAGTTTGTCACTGACT
GGAGAACTCGGTTTGTCGTCTGTTGCGGGGGCGGCAGTTATGCGGTGCCGTTGGGCA
GTGCACCCGTACCTTTGGGAGCGCGCGCCCTCGTCGTGTCGTGACGTCACCCGTTCT
GTTGGCTTATAATGCAGGGTGGGGCCACCTGCCGGTAGGTGTGCGGTAGGCTTTTCT

CCGTCGCAGGACGCAGGGTTCGGGCCTAGGGTAGGCTCTCCTGAATCGACAGGCGCC
GGACCTCTGGTGAGGGGAGGGATAAGTGAGGCGTCAGTTTCTTTGGTCGGTTTTATG
TACCTATCTTCTTAAGTAGCTGAAGCTCCGGTTTTGAACTATGCGCTCGGGGTTGGC
GAGTGTGTTTTGTGAAGTTTTTTAGGCACCTTTTGAAATGTAATCATTTGGGTCAAT
ATGTAATTTTCAGTGTTAGACTAGTAAA

RSV promoter sequence (229 bp)

AATGTAGTCTTATGCAATACTCTTGTAGTCTTGCAACATGGTAACGATGAGTTAGCA
ACATGCCTTACAAGGAGAGAAAAAGCACCGTGCATGCCGATTGGTGGAAGTAAGGTG
GTACGATCGTGCCTTATTAGGAAGGCAACAGACGGGTCTGACATGGATTGGACGAAC
CACTGAATTGCCGCATTGCAGAGATATTGTATTTAAGTGCCTAGCTCGATACATAAA
C

SV40 promoter sequence (351bp)

GGTGTGGAAAGTCCCCAGGCTCCCCAGCAGGCAGAAGTATGCAAAGCATGCATCTCA
ATTAGTCAGCAACCAGGTGTGGAAAGTCCCCAGGCTCCCCAGCAGGCAGAAGTATGC
AAAGCATGCATCTCAATTAGTCAGCAACCATAGTCCCGCCCCTAACTCCGCCCATCC
CGCCCCTAACTCCGCCCAGTTCCGCCCATTCTCCGCCCCATGGCTGACTAATTTTTT
TTATTTATGCAGAGGCCGAGGCCGCCTCGGCCTCTGAGCTATTCCAGAAGTAGTGAG
GAGGCTTTTTTGGAGGCCTAGGCTTTTGCAAAGATCGATCAAGAGACAGGATGAGGA
TCGTTTCGC

PGK promoter sequence **(555bp)**

TTGGGGTTGCGCCTTTTCCAAGGCAGCCCTGGGTTTGCGCAGGGACGCGGCTGCTCT
GGGCGTGGTTCCGGGAAACGCAGCGGCGCCGACCCTGGGTCTCGCACATTCTTCACG
TCCGTTCGCAGCGTCACCCGGATCTTCGCCGCTACCCTTGTGGGCCCCCCGGCGACG
CTTCCTGCTCCGCCCCTAAGTCGGGAAGGTTCCTTGCGGTTCGCGGCGTGCCGGACG
TGACAAACGGAAGCCGCACGTCTCACTAGTACCCTCGCAGACGGACAGCGCCAGGGA
GCAATGGCAGCGCGCCGACCGCGATGGGCTGTGGCCAATAGCGGCTGCTCAGCAGGG
CGCGCCGAGAGCAGCGGCCGGGAAGGGGCGGTGCGGGAGGCGGGGTGTGGGGCGGTA
GTGTGGGCCCTGTTCCTGCCCGCGCGGTGTTCCGCATTCTGCAAGCCTCCGGAGCGC
ACGTCGGCAGTCGGCTCCCTCGTTGACCGAATCACCGACCTCTCTCCCCAGGGGGAT
CCACCGGAGCTTACCATGACCGAGTACAAGCCCACGGTGCGC

β-globin promoter sequence (366bp)

GCTTTGCTTCTCAATTTCTTATTTGCATAATGAGAAAAAAAGGAAAATTAATTTTAA

CACCAATTCAGTAGTTGATTGAGCAAATGCGTTGCCAAAAAGGATGCTTTAGAGACA

GTGTTCTCTGCACAGATAAGGACAAACATTATTCAGAGGGAGTACCCAGAGCTGAGA

CTCCTAAGCCAGTGAGTGGCACAGCATTCTAGGGAGAAATATGCTTGTCATCACCGA

AGCCTGATTCCGTAGAGCCACACCTTGGTAAGGGCCAATCTGCTCACACAGGATAGA

GAGGGCAGGAGCCAGGGCAGAGCATATAAGGTGAGGTAGGATCAGTTGCTCCTCACA

TTTGCTTCTGACATAGTTGTGTTG

Fig. S2 CG dinucleotides content in the CMV and SV40 promoter

CMV promoter sequence (589bp)

TAGTTATTAATAGTAATCAATTACGGGGTCATTAGTTCATAGCCCATATATGGAGTT

CCGCGTTACATAACTTACGGTAAATGGCCCGCCTGGCTGACCGCCCAACGACCCCCG

CCCATTGACGTCAATAATGACGTATGTTCCCATAGTAACGCCAATAGGGACTTTCCA

TTGACGTCAATGGGTGGAGTATTTACGGTAAACTGCCCACTTGGCAGTACATCAAGT

GTATCATATGCCAAGTACGCCCCCTATTGACGTCAATGACGGTAAATGGCCCGCCTG

GCATTATGCCCAGTACATGACCTTATGGGACTTTCCTACTTGGCAGTACATCTACGT

ATTAGTCATCGCTATTACCATGGTGATGCGGTTTTGGCAGTACATCAATGGGCGTGG

ATAGCGGTTTGACTCACGGGGATTTCCAAGTCTCCACCCCATTGACGTCAATGGGAG

TTTGTTTTGGCACCAAAATCAACGGGACTTTCCAAAATGTCGTAACAACTCCGCCCC

ATTGACGCAAATGGGCGGTAGGCGTGTACGGTGGGAGGTCTATATAAGCAGAGCTGG

TTTAGTGAACCGTCAGATC

SV40 promoter sequence (351bp)

GGTGTGGAAAGTCCCCAGGCTCCCCAGCAGGCAGAAGTATGCAAAGCATGCATCTCA

ATTAGTCAGCAACCAGGTGTGGAAAGTCCCCAGGCTCCCCAGCAGGCAGAAGTATGC

AAAGCATGCATCTCAATTAGTCAGCAACCATAGTCCCGCCCCTAACTCCGCCCATCC
CGCCCCTAACTCCGCCCAGTTCCGCCCATTCTCCGCCCCATGGCTGACTAATTTTTT
TTATTTATGCAGAGGCCGAGGCCGCCTCGGCCTCTGAGCTATTCCAGAAGTAGTGAG
GAGGCTTTTTTGGAGGCCTAGGCTTTTGCAAAGATCGATCAAGAGACAGGATGAGGA
TCGTTTCGC
